# Inclusive HIV Prevention in South Africa: Reaching Foreign Migrant Adolescent Girls

**DOI:** 10.3389/frph.2021.629246

**Published:** 2021-05-20

**Authors:** Miriam Temin, Cecilia Milford, Mags Beksinska, Debbie Van Zyl, Jonathan Cockburn

**Affiliations:** ^1^Poverty, Gender, and Youth Program, Population Council, New York, NY, United States; ^2^MRU (MatCH Research Unit), Department of Obstetrics and Gynaecology, Faculty of Health Sciences, University of Witwatersrand, Durban, South Africa; ^3^Community Media Trust, Rondebosch, South Africa

**Keywords:** foreign migrant, South Africa, adolescents, determinants of HIV risk, HIV prevention, safe spaces, intersectionality and gender

## Abstract

Within South Africa's HIV epidemic, foreign migrant adolescent girls and young women (AGYW) face unique challenges in an environment typified by xenophobia and structural inequity. The intersection of age, gender, and migrant-related factors creates threats that may exacerbate their HIV risk, including discrimination, limited social capital, and economic dependency. This paper explores HIV-related determinants of risk from the perspective of foreign migrant AGYW who participated in a Girls' Club project implemented by Community Media Trust. Within clubs, foreign migrant AGYW met weekly with a female mentor to discuss HIV, safety planning, financial literacy, and other topics. Focus group discussions (FGDs) were conducted with club members and parents to learn about pressing challenges in a context characterized by early sexual debut, high rates of teenage pregnancy, and relationships typified by material exchange. FGDs addressed HIV risk factors such as social isolation and limited access to services, exacerbated by migrant-related stigma and discrimination and lack of identity documents. The foreign migrant AGYW appreciated the role of the Girls' Clubs and mentors in helping them overcome barriers to school and health services as well as building their social and other assets. FGD results indicate that HIV prevention in South Africa should prioritize action to address the specific determinants of foreign migrant AGYW's HIV risk, as well as inclusive policies that recognize migrants' heterogeneity based on gender and age.

## Introduction

South Africa has the largest HIV epidemic in the world, with an estimated 7.5 million people living with HIV (1). The country's economic and gender inequalities and its legacy of apartheid place young African women at the center of the HIV epidemic (2). Although there are promising signs of a reduction in HIV incidence among young women aged 15–24, with annual incidence rates declining from 2.1 to 1.5% between 2012 and 2017, female incidence and prevalence remain higher than males across all age groups (3). A number of campaigns have been designed and implemented with the goal of reducing adolescent girls' and young women's (AGYW) HIV incidence. One of these campaigns, the DREAMS Partnership, delivered a multi-sectoral package of interventions to reduce new HIV infections among AGYW in 15 high-prevalence countries by addressing multiple determinants of AGYW's risk (4)^1^.

National HIV campaigns do not explicitly target the large, heterogeneous population of foreign migrants living in South Africa. Although migrants to South Africa were historically made up of young men seeking work, the country has now become the regional migration hub in Southern Africa for women. Estimates indicate that the number of women migrants in South Africa has quadrupled since 1990 (5). Up to 4 million foreign migrants lived in South Africa in 2017 according to a UN Department of Economic and Social Affairs (UNDESA) estimate (6). Within this population, the large number of foreign migrant AGYW may face unique challenges above and beyond the broad challenges AGYW face in South Africa. A confluence of factors exposes many foreign migrant AGYW to regular discrimination, exploitation, economic dependency, and xenophobia. While adolescent-specific evidence is limited, focus group discussions carried out with 79 mixed female migrants in Cape Town indicated that all participants experienced xenophobia at both the community and official levels (7).

Intersectionality theory provides a useful framework for examining the ways that gender and age interact with migration status to shape HIV risk (8, 9). The interplay between migrant-specific issues and gender inequality within the dominant systems of structure and power may place foreign migrant AGYW at high risk of HIV infection along with other major risks. While evidence on the ways that mobility influences AGYW HIV risk is limited, research reveals more risk behaviors and higher HIV prevalence among migrant than non-migrant women in South Africa (10–12). A study of Zimbabwean women in Johannesburg uncovered low HIV risk perception, unresponsiveness of health workers, financial insecurity, and shaky legal status (13).

Reflecting their young age and migrant status, adolescent migrants face particular risks. These include factors like relatively low school enrolment; 66% of foreign-born adolescents attend school compared to 89% of South African adolescents (14). Migrant children from Zimbabwe have found it difficult to access schooling in South Africa and experience discrimination when in school (15). Unstable housing is also a concern; nearly three times as many foreign-born as South African adolescents live in informal dwellings (16). Factors like these may pose an additional threat to sexual and reproductive health (SRH). Illustrating this, twice as many foreign-born adolescents have given birth compared to South African adolescents (17).

Accessing reproductive health services remains a challenge to AGYW in South Africa more generally. Despite numerous attempts to implement targeted adolescent and youth-friendly services, no quality of care differences have been found to date between these targeted services and facilities not implementing specific services for AGYW (18–20). Although limited, the few studies exploring migrant and refugee's experience of accessing reproductive health services found that women experience “medical xenophobia,” which is defined as negative attitudes and practices of health sector professionals toward migrants and refugees in their work (21–23). In a study of health workers in South Africa, approximately 20% reported that they had witnessed discrimination and differential treatment of migrants in their workplaces (24). This is borne out by female migrants who report that health workers have criticized them and made them wait longer for health services than South African woman (25).

### The Girls' Club—An Intervention to Build AGYW's Social and Other Assets

The threats that foreign-born migrant AGYW face in a high-HIV prevalence context like South Africa influence their HIV infection risk. Reducing their vulnerability merits multisectoral action that addresses intersecting drivers of risk at multiple levels—individual, family, community, and political context. With the aim of addressing foreign migrants' risk in Durban and Johannesburg, Community Media Trust (CMT) implemented a Girls' Club project in the context of the DREAMS Innovation Challenge^2^. Clubs included foreign migrant AGYW aged 10 to 29 years old in eThekwini district of Durban and Johannesburg's City Center. In both locations, the program targeted foreign migrant girls by recruiting through foreign migrant community structures, migrant-serving organizations, and churches and schools with large proportions of foreign migrant participants.

The project aimed to build foreign migrant AGYW's social and other assets to address their exclusion, economic instability, and life skills in a protective setting. It enabled honest, open dialogue to build AGYW's knowledge, resilience, and coping strategies; it also built AGYW's social capital based on evidence that social isolation can be associated with HIV risk (26). Clubs met weekly with a slightly older female mentor from the local area who covered a curriculum on HIV and SRH, safety planning, and financial literacy. Following an initial phase when the program lasted 40 weeks, it was shortened to 20 weeks based on feedback that the commitment required was too lengthy.

Sessions provided AGYW with social support through regular meetings with peers and mentors who used participant-centered methods, like storytelling and role-play, to help AGYW absorb information and improve their skills. Mentors delivered a multisectoral curriculum covering: (1) Social Assets, such as being a good friend, engagement in the community, identity, and goals; (2) Cognitive Assets: such as safety plans, personal organization for school, communication skills, and ability to cope with xenophobia; (3) Health Assets, such as knowledge of body basics, puberty and menstruation, HIV, and family planning; and (4) Economic Assets, such as goals, savings, and knowledge of good and bad spending and borrowing wisely.

Through qualitative research methods, this paper explores the ways that being a young female migrant in South Africa can drive risk, including barriers and facilitators to uptake of SRH services. Risk factors are described from the perspective of foreign migrant AGYW and their parents who participated in Girls' Clubs. It describes how migrant status can exacerbate gendered HIV risks and the implications for programming and policy.

## Methodology

Foreign migrant AGYW and their parents participated in focus group discussions (FGDs) in Durban and Johannesburg, South Africa from December 2017 to December 2018. At the time of the FGDs, all AGYW had participated in or were currently participating in Girls' Clubs. The aim of the FGDs was to explore participant perspectives and experiences with the Girls' Club program and activities. As part of this, foreign migrant girls' experiences of HIV risk as well as mitigating influences including the Girls' Clubs were investigated.

### Study Population

Six FGDs were conducted with foreign migrant AGYW—three in Durban and three in Johannesburg. Twenty-five AGYW participated in Durban and 26 participated in Johannesburg. Participants were selected using purposive sampling based on their age (14–19 years), willingness to be part of the FGDs, ability to communicate in English, and parental consent. Participants were also required to be near the end of the Girls' Clubs program, having completed more than 75% of the sessions.

Two FGDs were held with parents of migrant girls in Durban (*n* = 10). Participating parents were also purposively selected. They had children who participated in the Girls' Clubs and were able to communicate in English.

### Data Collection and Analysis

FGDs were conducted by trained South African female research assistants who had extensive experience in conducting focus groups and interviews with adolescent and other vulnerable population groups. The groups were conducted in English because it was the language of instruction for the Girls' Clubs, and the AGYW spoke more diverse home languages than the FGDs could accommodate.

The focus group discussion guides covered topics including social support, protection, and access to health services and other key resources. All discussions covered AGYW in the general community and then probed specifically for issues affecting foreign migrant AGYW.

Discussions were audio recorded and transcribed verbatim. Transcripts were read, code lists developed iteratively from the data, and data were thematically coded. Key research objectives guided coding and analysis. A portion of the transcripts were double-coded to ensure data validity and reliability. All transcripts were coded by a senior researcher with over two decades of coding experience; trained research assistants double-coded a portion. Coders compared and discussed coding practices throughout the process. Once the data were coded, members of the research team who conducted the group discussions discussed preliminary research findings. NVivo v10 (QSR International) was used to facilitate data coding and analysis.

### Ethical Considerations

The study was approved by the University of the Witwatersrand's Human Research Ethics Committee (HREC, approval number 171005). All AGYW provided written consent or assent (if under 18 years) to participation; parental consent was obtained for AGYW under 18 years. All parents in FGDs provided written informed consent to participation. All participants consented for the discussions to be audio recorded.

## Results

The foreign migrant AGYW who participated in FGDs were broadly similar between Durban and Johannesburg, with a few notable differences (Table 1). The AGYW in Durban were slightly younger and much less likely to have completed primary school than the AGYW in Johannesburg. The majority in both cities were reportedly single/without a partner and from households with a source of income. All parents had completed secondary school or beyond and were married and earning an income. The largest group of AGYW and all participating parents hailed from Zimbabwe or the Democratic Republic of Congo. Ten foreign migrant AGYW were born in South Africa but identified as being a foreign migrant if one or both of their parents were foreign. The study population reflects the typical origin of migrants into South Africa, with most originating in neighboring SADC countries (27).

**Table 1:** Demographic details of foreign migrant AGYW and parents of foreign migrant AGYW.

	**Total foreign migrant AGYW (*n* = 51)**	**Durban AGYW (*n* = 25)**	**Johannesburg AGYW (*n* = 26)**	**Parents of migrant AGYW (*n* = 10)**
Age: mean years (range)	15.9 (14–19)	15.6 (14–19)	16.2 (14–18)	38.3 (31–55)
**Highest level of education n (%)**				
Primary incomplete	10 (19.6)	8 (32.0)	2 (8.7)	0
Primary complete	38 (47.5)	14 (56.0)	24 (92.3)	2 (20)
Secondary complete (matric)	3 (5.8)	3 (12.0)	0 (0.0)	4 (40)
Tertiary	0	0	0	4 (40)
**Relationship status n (%)**				
Single, no partner	37 (72.6)	18 (72.0)	19 (73.1)	0
Casual relationship	1 (2.0)	0 (0.0)	1 (3.8)	0
In a relationship (unmarried)	11 (21.6)	6 (24.0)	5 (19.2)	0
Married and living together	0	0	0	10 (100)
In a relationship and multiple partners	3 (5.8)	2 (8.0)	1 (3.8)	0
**Source of income n (%)**				
Yes	39 (76.5)	19 (76.0)	15 (57.7)	10 (100)
Sometimes	5 (8.6)	1 (4.0)	4 (15.4)	0
No	9 (17.6)	2 (8.0)	5 (19.2)	0
Did not answer / unsure	5 (9.8)	3 (12.0)	2 (7.7)	0
**Country of origin**				
Zimbabwe*	15 (29.4)	6 (24.0)	9 (34.6)	5 (50)
Democratic Republic of Congo (DRC)	12 (23.5)	6 (24.0)	6 (23.1)	4 (40)
South Africa*	10 (19.6)	3 (12.0)	7 (26.9)	0
Burundi	2 (3.9)	2 (8.0)	0	0
Zambia*	2 (3.9)	0	2 (7.6)	0
Angola	1 (1.9)	0	1 (3.8)	0
Ghana*	1 (1.9)	1 (4.0)	0	0
Kenya*	1 (1.9)	1 (4.0)	0	0
Malawi*	1 (1.9)	0	1 (3.8)	0
Not specified	6 (11.8)	6 (24.0)	0	1 (10)

* *English as the national language*.

Themes emerged from the data that relate to the known drivers of young females' disproportionate HIV risk in South Africa and the role of migrant status in exacerbating that risk. Among the determinants of HIV and other health risks that were discussed, two emerged as especially important for foreign migrant AGYW and their parents: xenophobia and gender inequality. These determinants intersect and influence SRH through several mechanisms identified in the discussions, including the following: (1) access to health services; (2) access to education; (3) early sexual debut and teenage pregnancy; (4) material exchange in relationships; and (5) access to social support. These are described in detail below.

### Access to Health Services for Young Female Migrants

Participants described various barriers to accessing SRH services linked to foreign migrant status, which could adversely impact AGYW's sexual health. Migrant AGYW offered various explanations for low family planning and HIV service uptake, including healthcare providers' attitudes toward them and fears about lack of confidentiality and stigma. While these barriers are experienced by AGYW in general, FGD participants described experiences indicating how foreign status may exacerbate these barriers.

*[A]t the clinic those sisters [….]! They judging [….]. They will be discussing your problems just in front of you, […] and then while, maybe your parent's friend comes in and they ask, “what was happening with the child?”, I am sure she will tell (AGYW, Johannesburg)*.

*[E]specially with foreign girls it is worse, when you go [to a clinic] and they actually they see your surname [….] once they know you are foreign, [….] they treat you so differently, […] you need to go to the hospital, but they don't go just because of the fact that they are foreigners, [….] and they know the treatment that you get when you are a foreigner [….]. So, people just end up not going to hospitals anymore because of that (AGYW, Durban)*.

A parent underscored the difficulty and discrimination that many foreign migrants experience when accessing healthcare services, reflective of high levels of xenophobia in South Africa.

*I gave birth at [name of hospital], I was told by a doctor “We hate you”, yet I'm […] signing papers that I'm going to the theater. What would you do? The doctor is telling you that “We don't want you here. You have spoiled our country. […]”. Is it me? “See, all of you, I think you are the ones who are filling up our hospitals”. We are left with hours to go to the theater and the doctor is telling me that “We hate you”. And now you are going to the theater. You are seeing the same doctor again. He's going to inject you. Are you going to wake up or not? […] The doctor is signing the papers for you to go and operate you, is telling you that, “We hate you. Do you pay tax?” I say, “My husband pay tax because he is working here” (Parent of migrant AGYW, Durban)*.

AGYW also cited the lack of identity documents as a barrier to accessing healthcare services for foreign migrants. One AGYW noted that she could not afford to pay for services, which was a requirement for people without South African identify documents.

*Let's say you are a foreigner, right? You want to get tested or […] treated, they will ask for documents, then if you don't have those documents, then you have to pay a sum of money, then you end up dying because of not having such (AGYW, Johannesburg)*.

### Access to Education for Young Female Migrants

The data describe how the interplay between gender, age, and migrant status also can pose barriers to education. AGYW described how regulatory and financial hurdles may limit migrants' school access, especially hurdles related to paperwork^3^. Foreign migrant AGYW reported challenges accessing identity documents, which were reported as a requirement for entry into some schools as described below.

*So we wrote [entrance] exams [for a particular high school] and then I was accepted […] and then they like “you need a South African passport for you to come to this school”. But when we researched the school you told us it was a school for what? For Africans, so African to South African, is South African not Africans? (AGYW, Durban)*.

*[I]t is not only about school fees, some of us, especially us migrant children […] we go to Home Affairs, they don't want to give us papers and at school they want papers before, before you can apply at school they want a paper. They ask. When we go to Home Affairs, they want money, they will tell us “you must come tomorrow” and they don't give us papers (AGYW, Johannesburg)*.

AGYW also described how they were stigmatized as foreigners by other learners within their schools and highlighted how difficulties understanding local languages posed additional barriers to learning.

*Being a foreigner is tough, in school if you have a weird surname they say “Aw Zayi zayi” [a common local word for foreigners] […] like they can even tease your surname in front of [an] important person (AGYW, Durban)*.

*For me [being in school was] very difficult because of first the language […], and when I would ask the explanation, the teacher always say “you go to this person, they will explain for you”, […] and when you go to the person, the person would say “No! Me I don't know, English is very hard for me to explain for you in English, […] I can explain for you in Zulu”, and you don't know Zulu, you must learn also Zulu, so there is no chance for you (AGYW, Johannesburg)*.

A parent described how their child experienced discrimination through being bullied due to their foreign status.

*I had a problem last year. [….] My daughter was in grade eight. […] And one day in her class there is one, one girl […] who called her “Happy kwerekwere!” [South African slang for foreigner]. And my daughter had a short temper and then she gave her what she was supposed to give [referring to physical violence] [laughter]. And then the teacher came “What happened!”? “Hey, she called me kwerekwere!”. And the child was down and she was bleeding on the nose. They called her parents and they called me too (Parent of AGYW, Durban)*.

### Early Sexual Debut and Teenage Pregnancy

Migrant AGYW reported that sexual debut happens early in their communities (between 12 and 15 years). Many viewed teenage pregnancy as common in their neighborhoods. One group of AGYW offered that teenage pregnancy was so common that it was normalized, and that it was even seen as desirable in their school.

*[I]n my school, pregnancy is such a common thing that you just see it as a daily job.[…] people are just there, “Let me follow my friend let me also get a belly”, […] They just [say] “let me upgrade to pregnancy” and they think it's such a good thing, “let me grow my tummy” […] They think it's like a new trend, you know, like a new style. “Let me also get some pregnancy, so then I am gonna be known as a woman…” (AGYW, Durban)*.

However, some AGYW reported that pregnant teenagers were the subject of discrimination in schools, which, in turn, impacted their access to education. They reported that some pregnant teenagers quit school due to shame and because of being poorly treated; others quit to get married.

*[A] girl in my school, many kids talk about her behind her back, ‘cause she’s pregnant [….] They get treated like dirt (AGYW, Johannesburg)*.

*They drop out [of school] because they are afraid that they will be judged at school (AGYW, Johannesburg)*.

Parents of AGYW also discussed the rise in teenage pregnancy. They associated it with substance use practices like drinking alcohol and smoking, which they felt led to poor decision-making and loss of self-control. The parents emphasized the importance of providing AGYW pregnancy prevention options such as condoms.

*[T]hese days girls are drinking more than even boys. They are smoking more than boys, which was reversed before. Now [mobile clinic staff] were telling [AGYW], “You see what you are, you are putting yourself in? Drinking. You can lose your control and then a boy can take advantage on you and then go to sleep with you without any [prevention methods] or condoms, and then you fell pregnant. Please, even your drinking, there is some packets [condoms] here. Take it. Don't, don't shy to take it” (Parent of AGYW, Durban)*.

Parents described the difficulty of discussing sensitive topics with their children and expressed the wish that their children would learn about topics related to sex from the Girls' Club mentors.

*I wish [the mentors] should be straight-forward and call a spade a spade when they speak with these children. […] When [my daughter and I] are sitting together, I end up getting scared to speak some words because I tell myself that, eish, maybe I do something wrong. But since she joined this programme, I am relieved because I know that they are going to touch on this issue. Although I have said it, but I said it half-way [only told her daughter a bit of information] (Parent of AGYW, Durban)*.

### Material Exchange in Relationships

Most of the migrant AGYW expressed that it was common for AGYW to be in sexual relationships with men who, in return, provide them with financial or other material gain. They described how these types of relationships may limit AGYW's power and expose them to sexual health risk, exacerbating the effects of gender inequality among these groups. Some AGYW justified these types of transactional relationships and felt that they were necessary to improve their current family and/or living situations.

*[W]hen your family is not […] well known financially and […] you don't have the means to survive on a daily basis, and then there is this rich guy who is coming who wants to support you and he's showing you the bright side […]. We tend to follow that side ‘cos we seeing that […] “if I go there I can help my family”, which is wrong, […] They see that “if I can be with this guy I am not doing something wrong, I am doing this so that I can help my family”, maybe that is the reason they use, that is very common (AGYW, Durban)*.

Some foreign migrant AGYW noted instances when there was parental support for relationships with older men. They described how, in some cases, parents encouraged early marriage for both financial and cultural reasons, especially for those who originated in contexts where child marriage is accepted.

*[S]ome of the parents don't really care, so they actually encourage them to go on and do it [have relationships for financial gain] (AGYW, Durban)*.

*[S]ometime[s] parents will, […] some, people get married early. […] So, sometimes it's based like on culture that you should get a man that could provide for you, and that can take care of you (AGYW, Johannesburg)*.

A few migrant AGYW proposed alternatives to inequitable transactional relationships. They suggested the importance of aspiring for a better future or seeking employment opportunities instead, which, they noted, was something they learned from participating in a Girls Club.

*[W]e growing up in a world where everything is instant, instant food, instant data, instant banking, so when people […] not getting an instant answer, then they want to use their own methods and then that's we when we end up going to blessers*^4^. *[…], but […] there are places out there that offer jobs […], it's a matter of knowing your self-worth like how the [Girls] club teaches […] of your self-worth, setting goals for yourself, being self-motivated, ‘cause when you know what you worth you will never let anybody put your worth any lower than your self-worth (AGYW, Durban)*.

### Access to Social Support

The data describe how the interplay between gender, age, and migrant status can socially isolate AGYW and the vital importance of social support for them. AGYW reflected on the lack of close friendships among peers, which migrant status can exacerbate. Some described the difficulty they had trusting others, which affected their ability to open up and develop relationships with their peers, resulting in many bearing burdens on their own.

*Cause trust […], just the word itself trust, hmm mm [disagrees] it hard for me to trust somebody. Yes, I can you trust you with all the light things but when it comes to the heavy things, […] I feel it not easy and all (AGYW, Durban)*.

*What I like about this club is that immigrant um girls, like foreigner, foreign girls, they are discriminated by the, the country where they come from but this, this club everyone is equal no matter where you come from, and they don't judge us (AGYW, Johannesburg)*.

Many AGYW also characterized parent–child relationships as mistrustful, describing their parents' suspicions about their behavior. The following quote reflects how gender norms can shape their relationship with their parents.

*[P]arents don't trust their children. Every time the child goes out they have that mentality that person is going to do something bad outside and […] embarrass ourselves […]. My mother, [….] she always has that thing “she's a girl and girls get naughty sometimes so she will do something bad” (AGYW, Johannesburg)*.

The AGYW described how the Girls' Club Project created safe spaces for them to build relationships with peers. FGD participants appreciated the opportunity that the Girls' Clubs provided for them to discuss issues in a non-judgmental environment with other foreign migrant AGYW.

*I think instead of saying we have made friends, actually we all have become sisters, we have put aside all our difficulties and we have just became like one, cause every time we meet all we do is just laugh and learn, laugh and learn, it's like you want it to be happening every day. […] And you wish you were living together, [….] that's how good it has become (AGYW, Durban)*.

*What I like [most] about this club is that immigrant girls, […], foreign girls, they are discriminated by the, the country where they come from but this, this club everyone is equal no matter where you come from, and they don't judge us (AGYW, Johannesburg)*.

The trusting relationships AGYW built with their mentors in particular became a source of social support. Many AGYW described the trusting relationships they developed with their group mentors:

*[T]hey are like diaries for us, you can tell them anything and they will keep it for you. They take your problems like their own, you see, so it feels nice to have them (AGYW, Johannesburg)*.

Migrant AGYW described the positive influences that their mentors had on their lives, for example, teaching them how to develop their confidence, resilience and coping strategies, and how to make friends.

*[S]o Girls Club built up our confidence, resilience, and tells us more things that are happening around […]. So it's good to come to Girls Club cause it teaches you how to be careful and know, who you talk to and to choose your friends wisely (AGYW, Durban)*.

The AGYW described how they were able to discuss things with mentors that they couldn't discuss with parents, such as life skills and coping strategies.

*I think it is actually the best thing to have a mentor […] Like they know the inner part of you that your parents don't know, and they actually know how you feel in all of your stages that you go through (AGYW, Johannesburg)*.

A parent reinforced the value AGYW placed in the trusting relationships they built with mentors, and of the bond that they created.

*My daughter […] they used to like their mentor a lot. […] I think they are free to their mentor. […] they have developed like a bond with their [mentor] […] they are so close and friendly. And she's so kind (Parent of migrant AGYW, Durban)*.

## Discussion

The FGDs shed light on young females' disproportionately high HIV risk in South Africa and, in particular, how gender and age can intersect with foreign migrant status to exacerbate risk in key areas. Foreign migrant AGYW may face disadvantages reflecting xenophobia and gender inequality, which have both direct and intermediary influences on HIV-related behaviors and outcomes. Direct influences included barriers to health services and inequitable relationships anchored in material exchange, which play important roles in sustaining South Africa's HIV epidemic (28). Intermediary influences included barriers to schooling, economic instability, and limited social support.

The AGYW described unreliable friendships and mistrustful relationships with parents, exacerbated by their foreign migrant status, which is concerning in light of evidence on the association between social capital and risk. Research elsewhere in South Africa found that social isolation was associated with a higher risk of early pregnancy. It also found that social capital can be protective; girls' participation in sports groups and trust in their neighbors were associated with increased condom use (29). The AGYW in these Girls' Clubs described how their participation enabled them to develop trusting relationships with their mentors, building their own social capital.

While several of the determinants of risk discussed by the foreign migrant AGYW overlap with the determinants of risk for South African AGYW, it is vital to consider how migrant status intersects with age and gender to amplify their HIV risk. Their young age, female gender, and foreign migrant status place these AGYW at the nexus of interrelated challenges in a context of xenophobia and systemic stigma (30). For instance, the FGDs provided a platform to highlight concerning health service and school-related barriers associated with the lack of identity documents. While insufficient paperwork is not unique to foreign migrants, language and stigma and discrimination can pose additional barriers to their access to essential services.

Programming to build foreign migrant AGYW's protective social capital and other assets, resilience, and sense of self-worth provided an opportunity to address some of these factors, which can address the determinants of risk discussed above. AGYW in Girls' Clubs valued the opportunity to meet regularly with mentors and peers who were similar to them in a safe, non-judgmental environment. Furthermore, they appreciated the trusting relationships they developed with their mentors, who served as role models and motivated group members' aspirations, coping skills, and resilience. AGYW cited mentors' roles in providing emotional support in the face of social isolation, potentially addressing a key HIV risk factor. This project demonstrated the potential of locally recruited mentors to build AGYW's assets and help them develop skills and agency to put their assets to use.

An evidence review of community-based girls' groups found that most effects relate to individual participants' attitudes, beliefs, knowledge, and awareness, while platforms like Girls' Clubs also offer opportunities to help AGYW overcome barriers to essential services (31). For example, in South Africa, gaining identity papers requires information and courage to access the Department of Home Affairs. In the Girls' Clubs, mentors shared accurate information and linked Club members to sources of assistance with accessing paperwork, which has the potential to benefit entire families. Mentors also sought to improve access to health services through active referrals and “opportunity visits” to clinics with groups of AGYW to help overcome fear; mentors also shared their own experiences to motivate Club participants. The project further aimed to build AGYW's economic assets including financial literacy, which can help them navigate economic instability.

In addition to the discrimination and fear that many foreign migrants face as a result of xenophobia and systemic threats, these challenges can limit migrant families' livelihood opportunities (32). Poverty and economic instability are important drivers of inequitable transactional relationships and the inherent HIV transmission risk they carry (28). While strengthening foreign migrant AGYW's livelihood opportunities was outside of the scope of the Girls' Club project, skills-building, small business training, and other forms of economic empowerment could play a role in creating alternatives to transactional relationships. This may have the potential to disrupt an important risk pathway for foreign migrant AGYW.

## Limitations

There are some limitations to the interpretation of our results. Due to the qualitative, exploratory nature of this research, results may not be generalizable nor may they be representative of all foreign migrant AGYW in urban South Africa given that participants were all members of Girls' Clubs. Furthermore, no FGDs were conducted with comparison groups such as native South African AGYW and their parents who had attended Girls' Clubs, or foreign migrant AGYW and their parents who had not attended Girls' Clubs.

To note, FGD moderators probed about specific issues experienced by foreign migrant AGYW, which we attempted to highlight, although discussions covered general issues common to many AGYW in South Africa (whether foreign migrant, attending Girls' Clubs, or not). Finally, a few participants had trouble understanding complex descriptions in English; while others in the groups translated in such cases, limited English ability could have impacted the depth of descriptions.

## Implications and Conclusion

The data presented here underscore the need for more inclusive HIV prevention programming that reaches foreign migrant AGYW. To be effective, programming should be responsive to the direct and intermediate determinants of risk that reflect the intersection of factors threatening their health and well-being. In the Girls' Club project, regular group meetings with mentors and peers appeared to be a powerful platform to address some of the intersecting challenges of being young, female, and foreign in an environment characterized by xenophobia and discrimination. The mentors who served as role models and inspired alternative future aspirations were a vital element of this program model. Interventions to reduce barriers to essential services by targeting attitudes and behaviors of healthcare providers and immigration and school authorities are also priorities, along with opportunities to improve migrants' livelihoods. The impact of multisectoral programming for AGYW can be amplified by multilevel action to address the determinants of risk among families, communities, and institutions.

While individual benefits may contribute to reducing foreign migrant AGYW's risk, broader action is needed to deal with systemic stigma, xenophobia, and structural barriers that exacerbate and sustain risk. Beyond more programming, migrant health and well-being merit policy-level attention that is sensitive to migrants' heterogeneity based on gender and age. Policies also need to recognize and address the legal context, the perspectives of the state, and debates around illegal or undocumented migration in wider society. In particular, first, migrants must be included in the context of HIV prevention and treatment policies and planning (33). Second, formal policies should take account of migrant access to health services and prohibit healthcare workers from violating migrants' needs and rights to reduce a key barrier to services. Third, policies and services should ensure foreign migrant AGYW's access to schooling for the health, social, and other benefits.

## Data Availability Statement

The raw data supporting the conclusions of this article will be made available by the authors, without undue reservation.

## Ethics Statement

The studies involving human participants were reviewed and approved by Human Research Ethics Committee (Medical) of the University of the Witwatersrand. Written informed consent to participate in this study was provided by all participants aged 18 or older. Where participants were under 18 years, written consent was provided by the participants' legal guardian/next of kin, and they provided written assent. All participants consented to audio recording.

## Author Contributions

CM and MT participated in the planning and coordination of the study with MB advising. CM, DV, MB, and JC coordinated the data collection. CM and MB participated in the data analysis. CM and MT led the write-up of the results with review. Final approval of the final manuscript by MB, DV, and JC. All authors contributed to the article and approved the submitted version.

## Conflict of Interest

The authors declare that the research was conducted in the absence of any commercial or financial relationships that could be construed as a potential conflict of interest.

## Footnotes

^1^DREAMS is a global partnership between the U.S. President's Emergency Plan for AIDS Relief (PEPFAR), Bill & Melinda Gates Foundation, Girl Effect, Johnson & Johnson, ViiV Healthcare, and Gilead.

^2^This work was funded by the DREAMS Innovation Challenge, which supported projects like the Girls' Club project with different target populations outside the original DREAMS scope.

^3^The South African Department of Education requires all learners who take grade 12 (matric) exams to have a South African identity document and number as proof of identity for writing their exams.

^4^“Blesser” is a slang term for a rich man who offers support (typically financial and material) to a younger female companion in exchange for sex, friendship, etc., https://www.urbandictionary.com/define.php?term=blesser.
