# Supplementary material for: Inclusive HIV Prevention in South Africa: Reaching Foreign Migrant Adolescent Girls
Source: Front Reprod Health. 2021 May 20;3:629246. doi: 10.3389/frph.2021.629246 (PMC9580656; doi:10.3389/frph.2021.629246)
Supplement: Supplementary file 1 [file Data_Sheet_1.PDF]

MatCH Research Unit
Department of Obstetrics and Gynaecology
Faculty of Health Sciences
University of the Witwatersrand

Focus group guide

Focus Group Identification Number:	
Facilitator:	
Note taker:	
Date of Focus Group (date/month/year):	
Start Time:	
Stop Time:	
Recorder Check Performed by:	
FGD with AGYW or parents of AGYW:	
FGD in Johannesburg or Durban:	

Note to facilitators: *For the optimal use of this tool it is important to read through the tool carefully and prepare all the equipment required (i.e. index cards, markers, flipcharts etc.) prior to the start of the focus group discussion. Conducting this focus group well will yield results which are core to the success of the overall project.*

Facilitator Instructions:

- *Ask each main question.*
- *The follow-up questions are to help you obtain more information from the participants and encourage them to talk more specifically about their experiences.*
- *General probes can be used encourage more discussion. Examples of general probes are:*

o *"Please tell us more about that."*

o *"Please give us an example."*

o *"How do others feel about that?"*

o *"Has anyone had a different experience?"*

Co-Facilitator / note taker Instructions:

Note participants who attempt to speak but are interrupted or who otherwise have difficulty entering the discussion, and facilitate their contribution by saying "I believe [participant name as presented on name card/tent placed in front of participant] was about to speak. Please go ahead," or a similar phrase. Monitor the facilitator's progression through the guide to ensure that all topics are addressed.

Ground rules for the discussion

- *Introduce the study and purpose of the FGD*
- *Welcome the participants and thank them for having agreed to participate in this FGD.*
- *The opinion of each of you is important for us and there's no right or wrong answer.*
- *We would like our discussion to be organized so we can hear each other without interruption.*

- *We will be tape/ digitally record this FGD so we can accurately document every word. We would like to assure you that no one will hear these recordings except the monitoring and evaluation team and no names will be mentioned in the final report.*
- *We would like each one of you to speak clearly and wait for your turn.*
- *It is of utmost importance that each of you respects the confidentiality of all what is mentioned during this FGD. You don't need to mention your true or full name during the discussion. Any information discussed during this focus group should not be disclosed outside the group.*
- *Do you have any questions before we start?*

Introduction and Description of Project:

Hello, and thank you for agreeing to participate in this focus group discussion. My name is ______________, and I am the facilitator for this group. [Introduce colleagues, note-takers, etc].

The purpose of a focus group is to learn about your ideas and opinions. We are inviting you to take part in a discussion about your experiences with the CMT DREAMS Girls Clubs Program. We would like you to share your understanding of young people's views and insight into the acceptability, relevance, and impact of the program activities for other young women. The discussion will last 2 – 2.5 hours.

Ngiyabingelela, ngiphinde ngibonge ngokuvuma ukubamba iqhaza kwinqxoxo yeqembu ehleliwe. Igama lami ngingu____________________ , futhi ngingumphathi walengxoxo yaleliqembu. [yazisa osebenza nabo, obhala amanothi, nokunye]. Inhloso yengxoxo yeqembu ukufunda mayelana nemicabango kanye nemibono yenu. Siyakumema ukubamba iqhaza kwingxoxo mayelana nosuhlangabazane nakho kuhlelo lwe lwe-CMT DREAMS Girls Clubs. Singathanda ukuthi wabelane nathi ngokuqonda kwakho ngemibono yabantu abasha kanye kanye nemibono yokwamukela, nokuqondene, kanye nemithelela yezinhlelo zohlelo kubanye abantu besifazane abasebancane. Ingxoxo izothatha isikhathi esingangamahora amabili kuya kwabili nohhafu (2-2.5 hours).

Social Support and Protection -ALL participants – 20 minutes	
**Lead questions**	**Secondary questions and probes**
**1.1 Tell us why you decided to take part in the CMT DREAMS Girls Clubs program?/**<br><br>***Sitshele ukuthi kungani wakhetha ukubamba iqhaza kuhlelo lwe - CMT DREAMS Girls Club?***	• What do you like about taking part in the Girls Clubs?/ *Uthandani ngokubamba iqhaza kwiGirls Club?*<br>• What are some of the main reasons that girls and young women come and participate?/ *Yiziphi ezinye izizathu okuyizonazona ezenza ukuthi amantombazane kanye nabesifazane abancane afike abambe iqhaza?*

**1.2 What do you like the most about taking part in group activities in the CMT DREAMS Girls Clubs program? /**<br><br>***Ikuphi okuthande kakhulu mayelana ngokubamba iqhaza ngemisebenzi yeqembu lohlelo lwe - CMT DREAMS Girls Club?***	• Do girls and young women in the Club show up regularly? / *Ingabe abesifazane abancane bayafika njalo kuleli qembu?*<br>• What would you improve or change about the Girls Clubs? / *Yini ongayishintsha noma ongayithuthukisa nge Girls Club?*
**1.3 Are parents or caregivers supportive of you attending the Clubs? /**<br><br>***Ngabe abazali noma abanakekeli bayakweseka ukuya kwakho eqenjini?***	• Do parents or caregivers support girls' and young women's regular participation in the Girls Clubs?/ *Ingabe abazali noma abanakekeli basekela abesifazane abancane kanye namantombazane abancane ngokuthi babambe iqhaza njalo kwiGirls club?*<br>• Or ensure that girls and young women use any of the groups or activities they learn about? / *Noma ukuqinisekise ukuthi amantombazane kanye abesifazane abancane basebenzisa noma iliphi iqembu noma imuphi umsembenzi abafunde ngawo?*<br>• Probe for differences between disabled AGYW and migrant AGYW / *Buzisisa umehluko phakathi kwaba khubazekile kwi AGYW kanye nabokufika kwi AGYW*
**1.4 Do you think other parents would want their daughters to participate in the groups? /**<br><br>***Uyacabanga ukuthi abanye abazali bangafuna ukuthi amadodakazi abo abambe iqhaza kuleliqembu?***	• What are some of the reasons that parents or caregivers might want AGYW like you to participate? / *Yiziphi ezinye zezizathu abazali noma abanakekeli abangase bafune iAGYW enjengawe ukuthi ubambe iqhaza?*<br>• What are some of the reasons that parents or caregivers might not let AGYW participate? / *Yiziphi ezinye zezizathu ezingenze abazali noma abanakekeli bangavumi ubambe iqhaza kwiAGYW?*<br>• Probe for disabled AGYW and migrant AGYW / *Buzisisa abakhubazekile kwiAGYW kanye nabokufika kwiAGYW*
**1.5 Are girls and young women facing any other obstacles or challenges to participating in the groups or following up with any of the groups or activities they learn about there? /**<br><br>***Ingabe amantombazane kanye nabesifazane abancane babhekene nezithiyo noma izinqinamba ngokubamba iqhaza kunama imaphi amaqembu noma ukulandelela amaqembu noma imisebenzi abayifunde lapho?***	• Are girls and young women facing any other obstacles or challenges with regards to attending the groups and or undertaking activities they learn about in the program? / *Ingabe amantombazane noma abesifazane abancane babhekene nanoma yiziphi enzinye izinselelo noma izithiyo ngokuqondene nokuya emaqenjini noma ukwenza imisebenzi abayifundayo mayelana nohlelo?*<br>• Probe for disabled AGYW and migrant AGYW/ *Buzisisa abakhubazekile kwiAGYW kanye nabokufika kwiAGYW*

**1.6 Have you made new friends with any of the other young women participating in the groups? /**<br><br>***Ngabe ubenzile ubungani obusha nanoma ibaphi abesifazane abancane ababambe iqhaza kuleliqembu?***	• Have you met new people in the group who you could confide in? / *Ingabe uhlangane nabantu abasha eqenjini ongavulelana nabo isifuba?*<br>• Have you met new people in the group who you could visit or ask for help in an emergency? / *Ingabe uhlangane nabantu abasha eqenjini ongavakashela noma ucele usizo kwisimo esiphuthumayo?*
**1.7 During groups young women are encouraged to work with a mentor. We would like to discuss how you and other young women in the groups feel about this./**<br><br>***Ngesikhathi seqembu abesifazane abancane bayakhuthazwa ukusebenzisana nomqeqeshi.Singathanda ukuxoxa ngokuthi wena kanye nabanye abantu besifazane abangcane kuleliqembu ukuthi niphatheka kanjani ngalokhu?***	• Do girls go to their mentor to talk about their own problems? / *Ingabe bayaya abasefazane kubaqeqeshi babo bakhulume ngezinkinga zabo?*<br>• Are these mentors available if anyone from the group needs to talk to her outside of the regular meeting times? / *Ngabe abaqeqeshi bayatholakala uma noma ubani ovela eqenjini adinga ukukhuluma naye ngaphandle kwezikhathi ezivamile zomhlangano?*<br>• Do all Club members feel encouraged to participate in the sessions by mentors / *Ingabe wonke amalunga eqembu azizwa ekhuthazwa ukuba ahlanganyele kumaseshini abaqeqeshi?*<br>• Do mentors ensure that everyone is treated equally? / *Ingabe abaqeqeshi bayaqinisekisa ukuthi wonke umuntu uphathwa ngokulinganayo***?**<br>• Probe for disabled AGYW and migrant AGYW / *Buzisisa abakhubazekile kwiAGYW kanye nabokufika kwiAGYW*

Access to Key Resources and Protection – All participants – 40 - 60 minutes	
**Lead questions**	**Secondary questions and probes**
**2.1 Are there organizations in your community that provide assistance for families who come to South Africa from other countries? /**<br><br>***Zikhona izinhlangano emphakathini wakho ezisiza imindeni ezeMzansi Afrika evela kwamanye amazwe?***	• Do you know of anyone who has received assistance from these organizations? / *Ukhona omaziyo owathola usizo kulezi zinhlangano?*<br>• Do these organizations help girls and young women? / *Ingabe lezinhlangano ziyawasiza amantombazane noma abesifazane abangcane?*<br>• If so, in what ways have they helped them, for example, do they help with providing ID documentation or papers? / *Uma kunjalo, ingabe babasiza ngayiphi indlela, isibonelo, ingabe bayasiza ngokunikeza amadokhumenti ama-ID noma amaphepha?*<br>• Are these organisations at school or in the community? / *Ingabe zikhona lezinhlangano ezikoleni noma emphakathini?*<br>• Probe for disabled AGYW and migrant AGYW / *Buzisisa abakhubazekile kwiAGYW kanye nabokufika kwiAGYW*
**2.2. What kinds of health and HIV services do girls and young women use in your community? /**<br><br>***Hlobo luni lwezinsiza lwezempilo kanye nesandulela ngculasi olusetshenziswa abantu besifazane abangcane kanye namantombazane emphakathini wakho?***	• Are these services used by young girls in your community? / *Ingabe lezinsiza zisetshenziswa abasefazane abancane emphakathini wakho?*<br>• Probe for migrant girls / *Buzisisa abesifazane bokufika*<br>• Probe for disabled girls / *Buzisisa abesifazane abakhubazekile*
**2.3. Before joining the program did you know about HIV or family planning services? /**<br><br>***Ngaphambi kokungenela loluhlelo ingabe wawu nawo ulwazi ngezinhlelo ngesandulela ngculazi noma izinsiza zokuhlela umndeni?***	• Did you or anyone you know access HIV or family planning services before the program? / *Ingabe wena noma ubani wakwazi ukuthola izinsiza zegciwane lesandulela ngculazi noma ezokuhlela umndeni ngaphambi kwaloluhlelo?*<br>• Has the program helped you and other young women connect with services, such as HIV testing, family planning, or other sexual and reproductive health services? / *Ingabe loluhlelo lusize ukuthi wena kanye nabanye abesifazane abancane ukuthi nixhumane nezinsiza, njengokuhlolela igciwane lesandulela ngculaza, ukuhlela umndeni, noma ezinye izinsiza zokuzalanisa kwezempilo. Noma ezinye izinsiza zezempilo zocansi nezokuzalanisa?*<br>• Probe for disabled AGYW and migrant AGYW / *Buzisisa abakhubazekile kwiAGYW kanye nabokufika kwiAGYW*

**2.4 Are most girls and young women in your community in school? /**<br><br>***Ingabe iningi lamantombazane noma abesifazane abancane bayafunda emphakathini wakho?***	• Are young women able to attend their classes regularly? / *Ingabe abesifazane abancane bayakwazi ukuya ezikoleni zabo njalo njalo?*<br>• What difficulties do they have attending classes? / *Yiziphi izinkinga abahlangabezana nazo uma beya emakilasini?*<br>• What helps them to attend classes? / *Yini ebasiza ukuba bafunde amakilasi?*<br>• Probe for disabled AGYW and migrant AGYW / *Buzisisa abakhubazekile kwiAGYW kanye nabokufika kwiAGYW*
**2.5 Have you had problems or do you know of young women who have had trouble enrolling in school /**<br><br>***Ingabe ube nazo izinkinga noma uyabazi abesifazane abancane ababe nezikinga zokubhalisa esikoleni ?***	• Were the students being difficult? / *Ingabe abafundi ababebanika inkinga?*<br>• Or principals or teachers? / *Noma uthisha nhloko noma othisha?*<br>• In what ways were the school environment welcoming or unwelcoming to you or other young women? / *Ingabe ngaziphi izzindlela izakhiwo zesikole ezenza wena noma abanye abesifazane abancane bazizwe amukelekile noma bangamukelekile?*
**2.6 How did you or the young women you know of manage to overcome these problems? /**<br><br>***Ingabe wena noma abesifazane abancane obaziyo nakwazi kanjani ukunqoba lezizinkinga?***	• Ask about parents or adult community members advocacy on behalf of girls; / *Buza ngabazali noma amalunga amadala omphakathi akhuthazayo ekumeleni lamantombazane*<br>• Can you or do you / young women get help from Governing bodies / principals / teachers / poilice or refugee groups? / *Ungakwenza noma uyakwenza/ abesifazane abancane bathole usizo kuma governing body/uthisha nhloko/othisha/amaphoyisa noma iqembu lababaleki /iqembu lababaleki bokuhamba*
**2.7 Do you or others do any activities in the program that help with school work? /**<br><br>***Ingabe ikhona noma imiphi imisebenzi yohlelo eyenziwa nguwe noma abanye esiza ngomsebenzi wesikole khona izinhlelo enizenzayo ezinsiza emsebenzini wesikole?***	• Are there any activities you have done in the program that make you feel excited about going to school and your homework? / *Ingabe ikhona imisebenzi oyenzile ohlelweni olwenza uzizwa ujabule ngokuya esikoleni nasemsebenzini wakho wesikole wasekhaya?*

**2.8 Some activities in the groups focus look at budgeting and managing finances. We would like to know how you feel about these activities, are they useful / not useful? /**<br><br>***Eminye imisebenzi kulamaqembu abheka isabelomali kanye nokuphathe kwezimali.Singathanda ukwazi ukuthi uzizwa kanjani ngalemisebenzi, Ngabe zinosizo/ azinasizo?***	• Have you used the information you learned in the group about budgeting or safe savings? Any examples how you or young women have used the information/ *Ulisebensile ulwazi olunikezwe eqenjini mayelana nokubekwa kwesabelomali noma ngokugcina imalo yakho ngokuphephile? Ngabe zikhona noma yiziphi iziboneloukuthi wena noma abesifazane abangcane balusebenzise kanjani lolulwazi.*<br>• Are you or other young women saving more money than you did before the group meetings? / *Ingabe wena noma abanye besifazane abancane bonga imali eningi kunokwenza ngaphambi komhlangano weqembu?*<br>• Have you and other young women in the groups developed new financial goals since you joined the group, and do they seem achievable? Can you share some examples of these goals or dreams? / *Ingabe wena kanye nabanye nabesifazane abancane abakuleli qembu senike nathuthukisa izifiso ezintsha zezezimali kusukela ekungeneleni leliqembu, futhi kubonakala sengathi iphumelele? Ungabelana ngeziibonelo zalezizifiso noma amaphupho?*
**2.9 Do you think that girls and young women in your group are more or less able to deal with bullies? /**<br><br>***Ucabanga ukuthi ingabe amantombazane kanye nabesifazane abancane eqembini bayakwazi kakhulu noma bayakwazi kancane ukubhekana nabaxhaphazi?***	• Do you think the program can help young women learn skills that will help them speak up against bullying? (Probe for types of information / are these skills offered / not offered by the program) / *Ucabanga ukuthi loluhlelo lungasiza abesifazane abancane ukuba bafunde amakhono azobasiza ukuba bakhulume ngokumelana nukuxhashazwa? (Buzisisa ngezinhlobo zolwazi/ ingabe lamakhono ayanikezelwa/ awanikezelwa ohlelweni)*<br>• Do you think that girls and young women in your group are gaining skills that will prepare them to avoid bullying? (Probe for types of information / are these skills offered / not offered by the program) / *Ucabanga ukuthi amantombazane kanye nabesifazane abancane abaseqinjini lakho bayathola amakhono ezobalungiselela ukugwema ukuxhashazwa (Buzisisa ngezinhlobo zolwazi/ ingabe lamakhono ayanikezelwa/ awanikezelwa ohlelweni)*

**2.10 Have you heard about xenophobic acts that have occurred against migrants (people born outside of South Africa, now living in South Africa)? /**<br><br>***Ngabe usukewezwa ngezenzo zodlame lokucwasana ngokobuzwe ezike zenzeka kwabokufika (Abantu abazalwa ngaphadle kweningizimu Afrika, manje bahlala eningizimu afrika)?***	• Have you heard of anyone affected by any xenophobic acts? / *Uke wezwa ngomuntu wokufika othintekile ngodlame lokucwasana ngokobuzwe?*<br>• Do you have any thoughts about what girls and young women like you could do in these types of situations? / *Uyaba nemicabango ngokuthi amantombazane kanye nabesifazane abancane bangenzenjani kulezimo enzikanje?*<br>• Do any of the skills or techniques you cover in your groups, seem like they might be helpful to you or others? Which ones if so?/ *Ingabe inoma amaphi amakhono noma amasu ahlanganiswe eqenjini lakho, kubonakala sengathi kungase kukusizewena noma abanye? Imaphi wona uma kunjalo?*
**2.11 Would the police be likely or unlikely to help young women in this group or similar to you in the community if they needed help? /**<br><br>***Ngabe amaphoyisa angakwazi noma ngeke akwazi ukusiza abantu besifazane abangcane kuleliqembu noma abacishe bafane emphakathini uma bedinga usizo?***	• What are some reasons why young women would need help from police? / *Yiziphi ezinye zezizathu ezenza abesifazane abancane badinge usizo kumaphoyisa?*<br>• What are some reasons why young women do not get help? / *Yiziphi ezinye zezizathu ezenza abasifazane abancane bangalutholi usizo?*<br>• How do you think we can change this? Make it easier for young women to get help when they need it? / *Ucabanga ukuthi singayishintsha kanjani lento?Kwenze kuba lula ukuthi abesifazane abangcane bathole usizo uma beludinga?*
**2.12 Have you or your family been able to access help from the police if they had a problem? /**<br><br>***Ngabe wena noma umndeni wakho usuke wakwazi ukuthola usizo emaphoyiseni uma benekinga?***	• What are some reasons why family members would need help from police? / *Yiziphi einye izizathu ezenza amalunga amadala omdeni adinge usizokumaphoyisa?*<br>• What are some reasons why adult family members do not get help? / *Iziphi izizathu zokuthi kungani amalungu omndeni amadal angatholi usizo?*<br>• How do you think we can change this? Make it easier for everyone in the community to get help when they need it? / *Ucabanga ukuthi singayishintsha kanjani lento? Yenza kube lula kuwo womke umuntu emphakathini ukuthola usizo uma bewudinga?*

**ONLY ASK FOR DISABLED AGYW GROUPS**<br>**2.13 Do you know girls and young women who receive the South African government's disability grant in your community? /**<br><br>***Ingaba uyawazi amantombazane noma abesifazane abancane abathola isibonelelo sokukhubazeka sikahulumeni wase ningizimu afrika emphakathini wakho?***	• Where did they learn about the disability grant? / *Bafunda kuphimayelana ngesibonelelo sokukhubazeka?*<br>• Did you or other girls learn information about the grant in the program, such as who qualifies and how go about getting a grant? / *Ingabe wena noma abanye abesifazane ufunde ulwazi mayelana nesibonelo ohlelweni,njengokuthingubani ofanelekayo nokuthi angahamba kanjani ngokuthola isibonelelo?*<br>• Do you think young girls would apply for this grant if they were given this information? / *Ucabanga ukuthi abesifazane abangcane angayifaka isicelo salesi Sibonelo uma anikwa lolu lwazi?*
**2.14 Would a social worker be likely or unlikely to help young women in this group or similar to you in the community if they needed help? /**<br><br>***Ingabe usonhlalakahle angakwazi noma ngeke akwazi ukusiza abesifazane abangcane kuleliqembu noma abacishe bafane emphakathini uma bedinga usizo?***	• What are some reasons why young women would need help from social workers? / *Yiziphi ezinye izizathu ezenza abesifazane abangcane badinge usizo lwabasenbenzi bezenhlalakahle?*<br>• What are some reasons why young women do not get help? / *Yiziphi zezizathu ezenza abesifazane abangcane bangatholi usizo?*<br>• How do you think we can change this? Make it easier for young women to get help when they need it? / *Ucabanga ukuhti singiyishintsha kanjani lento? Yenza kube lula kuwo wonke umuntu wesifazane omngcane athole uziso uma bewudinga?*
Access to health services– All participants – 40 - 60 minutes	
**Lead questions**	**Secondary questions and probes**
**3.1 Would you say it is common or uncommon for adolescents and young women similar to this group to have sexual relationships with different men who give them money or goods or services, such as medical care, taxi ride/lift? /**<br><br>***Ungathi kujwaylekile noma akujwayelekile ukuthi Intsha kanye nabesifazane abancane abacishe bafane naleliqembu ukuthi babe nobudlelwane bezocansi nabesilisa abahlukene ababanika imali noma izimpahla noma usizo, njenge ukunakekela okwemithi, ukuhamba ngetekisi/ulift?***	• Around what age do young women begin to have these types of relationships? / *Abesifazane abangcane ingabe beqala beneminyaka emingaki ukuba naloluhlobo bobudlelwane?*<br>• Do you know / think they are able to leave the relationship if they want to, including if they are abusive? / *Ingabe uyazi/bacabnga ukuthi bayakwazi ukushiyaubuhlobo uma befuna, noma behlukumezwa?*<br>• Do adults and parents know about these relationships / *Ingabe abantu abadala noma abazali bayazi ngalokhu ubudlelwane?*<br>• Do parents approve or disapprove of these relationships? / *Ingabe abazali bayavuma noma abakuvumeli lobudlelwane?*

**3.2 Is it possible for adolescents and young women to avoid these types of sexual relationships to acquire things that they need or that they want? Is there some other way to get what they need/want other than through sexual relationship? /**<br><br>***Kungenzeka yini ukuthi Intsha kanye nabesifazane abangcane begweme lezinhlobo zobudlelwano bocansi ukuze bathole izinto abazidingayo noma abazifunayo? Ngabe ikhona enye indlela yokuthola abakudingayo/ abakufunayo ngaphandle kokuba nobudlelwano ngokocansi?***	• Why do you think it is possible or not possible? / *Kungani ucabanga ukuthi kungenzeka noma akunakwenzeka?*<br>• How could someone avoid these relationships if they wanted to? / *Umuntu angayigwema kanjani lobudlelwano uma ngabe ufuna?*<br>• Are adolescents and young women who avoid these relationships admired/ or are they bullied and picked on? Why do you think this is the case? / *Ingabe intsha noma abesifazane abancane abagwema lubuhlobo lobudlelwane bayathandwa/ noma bayaxhashazwa bayasukelwa? Kungani ucabanga ukuthi kunjalo?*
**3.3 What are some reasons why you or young women similar to this group tend to visit a health service (clinic?)? /**<br><br>***Ngabe iziphi ezinye izizathu ezenza wena nabanye abesifazane abangcane abacishe bafane naleliqembu bavakashe izinsiza zasemitholampilo?***	• Do they go if they suspect they have a sexually transmitted infection? / *Bayahamba uma besolwa ngokuba nesifo socansi?*<br>• Do they go to access family planning? / *Bayahamba ukufinyelela ukuhlela umndeni?*
**3.4 Now we are going to discuss some services that sexually active adolescents and young women might access at a clinic.**<br>**You do not have to disclose any personal information we just want to discuss these services in general and not single anyone out. From your activities in the group sessions and from your interactions with girls similar to this group in the community do you think young women know how to avoid pregnancy? /**<br><br>***Manje sezizo xoxa nggezinye izinsiza ezitholakala emtholampilo ngabesifazane abangcane nentsha abazimbandakanya nocansi.***<br>***Akudingekile ukuthi uze udalulule imininingwane eqondene ngawe ngqo ngoba sifuna ukuxoxisana ngezinsiza ngenjwayelo singabaluli muntu ngokuqondile. Kusukela emsebenzini yenu eyeqembu kanye nokuxhumana namantombazane acishe afane naleliqembu emphakathini ucabanga ukuthi abesifazane abasha bayazi ukuthi bangazigwema kanjani ukukhulelwa?***	• Do young women want to avoid pregnancy? Why do you this this is the case? / *Ingabe abesifazane abancane bayafuna ukugwema ukukhulelwa? Kungani ucabanga ukuthi kunjalo?*<br>• Are there any young mothers in your community? / *Ingabe bakhona omama abasha emphakathini wakho?*<br>• How are they treated? Why do you think this is the case? / *Baphathwa kanjani? Kungani ucabanga ukuthi kunjalo?*

**Talking about preventing pregnancy do young women in your community access… /**<br><br>***Ukukhuluma ngokuvimbela ukukhulelwa ingabe abasefazane abangcane emphakathini wakho bayafinyelela…***	• Condoms? / *Amakhondomu?*<br>• Do you think they know how to use condoms? / *Ucabanga ukuthi bayazi asetshenziswa kanjani amakhondomu?*<br>• Where can they learn this information? / *Bangalufunda kuphi lolulwazi?*<br>• Do you think they know when to use condoms? / *Ucabanga ukuthi bayazi ukuthi asetshenziswa nini amakhondomu?*<br><br>• **Do they access family planning? / *Bayafinyelela ekuhleleni umndeni?***<br>• Do you think they know how to use family planning?/ *Ucabanga ukuthi bayakwazi ukusebenzisa izindlela zokuhlelela ukukhulelwa Izindlela zokuhlela umndeni?*<br>• Where can they or have they learnt this information from? / *Bangakwazi kuphi noma balufunde kuphi lolulwazi?*<br>• Are there any myths or beliefs about family planning or condoms in your community? / *Ingabe zikhona izinto noma izinkolelo mayelana ngokuhlela umndeni noma amakhondomu emphakathini wakho?*<br>• Is there anything that would make using condoms and family planning difficult in your community? / *Ingabe kukhona okungenza kube nzima ukusebenzisa izinto zokuhlela umndeni noma ukusebenzisa amakhondomu emphakathini wakho?*
**Conclusion**<br>*We are almost finished. I just have one more question before we end/ Sesiyaqeda.Nginombuzo owodwa ngaphambi kokuthi siqede*	
What are your most important needs that still have not been sufficiently addressed?<br>*Do you have any questions for me about anything we have discussed? Is there anything else you would like to share?/*<br>*Iziphi izidingo zakho ezibalulekile kakhulu ezingakabhekelelwa ngokwanele?*<br>*Ingabe ukhona onemibuzo kimi nganoma yini esixoxe ngayo? Ingabe kukhona okunye ongathanda ukwabelana ngakho?*	

This is the end of the focus group discussion. Thank you so much for sharing your ideas with us. Do you have any questions, or is there anything that you would like to add before we end? If you have further thoughts about any of the issues we discussed today, please call Dr. Mags Beksinska, the South African Principal Investigator of the study, whose details are on the information sheet and consent form that you have been given. Possible ice-breakers – Facilitator to assess which would be best to use and order dependent on group dynamic and energy. Use between one and two ice-breakers as needed. /

Lokhu sekungukuphela kwengxoxo yeqembu.Ngiyabonga ngokwabelana ngemibono yenu nathi. Ingabe unemibuzo, noma Ngabe kukhona ofuna ukukwengeza ngaphandle ngaphambi kokuqeda? Uma uneminye imibono mayelana nanoma yiziphi izinkinga esixoxe ngazo namhlanje, sicela ufonele uDokotela Mags Beksinska, umphenyi oyinhloko yaseningizimu Afrika locwaningo, imininingwane yakhe etholakala kwipheshana lolwazi noma imvume nolwazi onikwezwe lona.

Suggestions for successful ice-breakers:

a. Set a time limit and stick to it. If participants enjoy the games they often wish to keep going. The recommended time limit is 5-10 minutes.
b. The facilitator should be positive, enthusiastic and energetic as you are setting the tone for group participation.
c. If one of the icebreakers are not effective due to the group dynamic, stop and try another one.

Ice-breaker one: End the Sentence

Write the start of a question on the board - My favourite colour is… and go around the room with each person finishing the sentence as fast as they can. After everyone mentions a colour you can go around the room with each person explaining why or you can ask another question (e.g. my favourite season is…).

Ice-breaker two: Icebreaker Questions

Use when you need to relax participants – ask only one question per ice-breaker activity from the list below:

1. If you were a vegetable, what vegetable would you be?
2. If you woke up tomorrow as an animal, what animal would you choose to be and why?
3. What's your favourite thing to do in the summer?
4. If you were an ice cream flavour, which one would you be and why?
5. What's the weirdest thing you've ever eaten?

Ice-breaker three: Drummer

Start your group by practicing a few rhythms. Tap a simple beat, repeatedly, and have group follow you. When you're ready, start with a simple beat and move along the group continuing your beat. The next person adds something different to your beat, and so on around the room. Encourage creativity, fun and a mixture of techniques.

Some options to explore:

a. Tapping with fingers only.
b. Slapping with whole palm.
c. Tapping with finger nails only.
d. Knocking with knuckles.
e. Banging with soft side of fist.
f. Three quick taps in one beat.

Ice breaker four: Communication and listening

Give each member of the group a sheet of white paper, ask them to close their eyes and follow your instructions carefully.

Instructions:

1. Fold the paper in half
2. Tear a corner of the paper off
3. Fold the paper in half again
4. Tear another corner of the paper off
5. Fold the paper in half again
6. Tear another corner

Ask everyone to open their eyes and unfold their paper, look at the different patterns created. This activity shows that we all experience and communicate differently but all are valid experiences.
