# Supplementary material for: Inclusive HIV Prevention in South Africa: Reaching Foreign Migrant Adolescent Girls
Source: Front Reprod Health. 2021 May 20;3:629246. doi: 10.3389/frph.2021.629246 (PMC9580656; doi:10.3389/frph.2021.629246)
Supplement: Supplementary file 2 [file Data_Sheet_2.PDF]

MatCH Research Unit
Department of Obstetrics and Gynaecology
Faculty of Health Sciences
University of the Witwatersrand

Focus group guide

Focus Group Identification Number:	
Facilitator:	
Note taker:	
Date of Focus Group (date/month/year):	
Start Time:	
Stop Time:	
Recorder Check Performed by:	
FGD with AGYW or parents of AGYW:	
FGD in Johannesburg or Durban:	

Note to facilitators: *For the optimal use of this tool it is important to read through the tool carefully and prepare all the equipment required (i.e. index cards, markers, flipcharts etc.) prior to the start of the focus group discussion. Conducting this focus group well will yield results which are core to the success of the overall project.*

Facilitator Instructions:

- *Ask each main question.*
- *The follow-up questions are to help you obtain more information from the participants and encourage them to talk more specifically about their experiences.*
- *General probes can be used encourage more discussion. Examples of general probes are:*

o *"Please tell us more about that."*

o *"Please give us an example."*

o *"How do others feel about that?"*

o *"Has anyone had a different experience?"*

Co-Facilitator / note taker Instructions:

Note participants who attempt to speak but are interrupted or who otherwise have difficulty entering the discussion, and facilitate their contribution by saying "I believe [participant name as presented on name card/tent placed in front of participant] was about to speak. Please go ahead," or a similar phrase. Monitor the facilitator's progression through the guide to ensure that all topics are addressed.

Ground rules for the discussion

- *Introduce the study and purpose of the FGD*
- *Welcome the participants and thank them for having agreed to participate in this FGD.*
- *The opinion of each of you is important for us and there's no right or wrong answer.*
- *We would like our discussion to be organized so we can hear each other without interruption.*

- *We will be tape/ digitally record this FGD so we can accurately document every word. We would like to assure you that no one will hear these recordings except the monitoring and evaluation team and no names will be mentioned in the final report.*
- *We would like each one of you to speak clearly and wait for your turn.*
- *It is of utmost importance that each of you respects the confidentiality of all what is mentioned during this FGD. You don't need to mention your true or full name during the discussion. Any information discussed during this focus group should not be disclosed outside the group.*
- *Do you have any questions before we start?*

Introduction and Description of Project:

Hello, and thank you for agreeing to participate in this focus group discussion. My name is ______________, and I am the facilitator for this group. [Introduce colleagues, note-takers, etc].

The purpose of a focus group is to learn about your ideas and opinions. We are inviting you to take part in a discussion about your experiences with the CMT DREAMS Girls Clubs Program. We would like you to share your understanding of young people's views and insight into the acceptability, relevance, and impact of the program activities for other young women. The discussion will last 2 – 2.5 hours.

Ngiyabingelela, ngiphinde ngibonge ngokuvuma ukubamba iqhaza kwinqxoxo yeqembu ehleliwe. Igama lami ngingu____________________ , futhi ngingumphathi walengxoxo yaleliqembu. [yazisa osebenza nabo, obhala amanothi, nokunye]. Inhloso yengxoxo yeqembu ukufunda mayelana nemicabango kanye nemibono yenu. Siyakumema ukubamba iqhaza kwingxoxo mayelana nosuhlangabazane nakho kuhlelo lwe lwe-CMT DREAMS Girls Clubs. Singathanda ukuthi wabelane nathi ngokuqonda kwakho ngemibono yabantu abasha kanye kanye nemibono yokwamukela, nokuqondene, kanye nemithelela yezinhlelo zohlelo kubanye abantu besifazane abasebancane. Ingxoxo izothatha isikhathi esingangamahora amabili kuya kwabili nohhafu (2-2.5 hours).

Social Support and Protection -ALL participants	
**Lead questions**	**Secondary questions and probes**
**1.1 Are parents and caregivers generally supportive of girls and young women's regular participation in CMT's Girls Clubs)?**<br><br>***Ingabe abazali nabanakekeli bajwayele ukusekela amantombazane nabesifazane abancane ngokubambaa iqhaza kwi CMT girls Clubs njalo?***	• Do parents and caregivers follow up on content discussed in the Clubs or any group activities that their daughters participate in through the program, for example:<br>• (a)Talking about what their daughters learn about at the Clubs<br>• (b) accessing support organizations or<br>• (c) accessing services such as family planning services?<br>• *Ingabe abazali nabanakekeli bayakulandelela okuqukethwe izihloko ebekukade kuxoxiswana ngazo kuma Clubs noma kunoma miphi imisebenzi yeqembu amadodakazi abo abamba iqhaza kuzo ngokohlelo, isibonelo:*<br>• *(a) Ukukhuluma ngokufundwe amadodakazi kuma Clubs*<br>• *(b) Ukufinyelela ezinhlanganweni zokwesekwa noma*<br>• *(c) Ukufinyelela ezinsizeni ezinjengokuhlelela ukukhulelwa*
**1.2 Are parents or caregivers involved in the program?**<br><br>***Ingabe abazali noma abanakekeli bayazibandakanya nohlelo?***	• Do parents and caregivers attend sessions or get letters explaining about the Clubs from their daughters? / *Ingabe abazali noma abanakekeli bayawahamba amasessions noma bayazithola izincwadi ezichaza ngama Clubs kumadodakazi abo?*<br>• Probe for differences between disabled AGYW and migrant AGYW/ *Buzisisa abakhubazekile kwiAGYW kanye nabokufika kwiAGYW*
**1.3 Do parents and caregivers have any concerns about girls and young women's participation in the groups (NAME OF PROGRAM)**<br><br>***Ingabe abazali nabanakekeli banakho noma ikuphi ukukhathazeka ngokubamba iqhaza kwamantombazane nabesifazane abasebancane emaqenjini (IGAMA LOHLELO)?***	• Could these concerns cause parents to stop allowing their daughters to participate in the program? / *Kungezeka ukuthi lezikhathazo zibange ukuthi abazali bamise imvume yabo ukuthi amadodakazi abo abambe iqhaza ohlelweni?*<br>• Can these concerns be addressed? / *Ingabe lezizikhathazo zingaqondiswa?*<br>• How best can program staff address these concerns? / *Bangaziqondisa kanjani kangcono abasebenzi bohlelo lezizikhathazo?*

**1.4 Do parents and caregivers have any positive experiences linked to their daughters or girls and young women in the community participating in the groups (NAME OF PROGRAM)**<br><br>***Ingabe abazali nabanakekeli banakho okuhle okuxhumene nokubamba iqhaza kwamadodakazi abo noma abasifazane emaqenjini omphakathi (IGAMA LOHLELO)***	• Are parents supportive of following up with any of the information that girls are learning about at the club or activities their daughters undertake through the Clubs? / *Ingabe abazali bayakweseka ukulandelela noma iluphi ulwazi olufundwa amantombazane kuma Clubs noma imisebenzi eyenziwa amadodakazi kuma Clubs?*<br>• What benefit do you see in young women participating in the program? / *Iyiphi inzuzo oyibonayo ngokubamba iqhaza kwamantombazane asasemancane ohlelweni?*
**1.5 Do your daughters discuss the program (INSERT NAME OF PROGRAM) with you?**<br><br>***Ingabe amadodakazi enu ayaxoxa ngohlelo nani? (FAKA IGAMA LOHLELO)***	• How best can program staff involve parents in the program? / *Ingabe abasebenzi bohlelo banga babandakanya kanjani kangcono abazali ohlelweni?*
**1.6 Have your daughters developed close relationships with their mentors who lead the groups?**<br><br>***Ingabe amadodakazi enu akhe ubudlelwano obusondelene nabaqeqeshi abahola amaqembu?***	• Have your daughters / AGYW participating in this program developed friendships with other AGYW in the group? / *Ingabe amadodakazi enu kanye nabesifazane abasebancane ababambe iqhaza kuloluhlelo bakhe ubungane namanye amantombazane kanye nabesifazane abasebancane eqenjini?*<br>• If so, have you noticed any changes in girls e.g. they spend more time with friends or participating in extra activities? / *Uma kunjalo, lukhona noma iluphi ushintsho olubonile emantombazaneni isibonelo basebenzisa isikhathi esiningi nabangani noma babamba iqhaza emisebenzini engeziwe?*<br>• Have they made new, trustworthy friends who they can rely on if they encounter problems or untrustworthy friends? / *Ingabe sebenze ubungane obusha futhi obuthembekile abangakwazi ukuthembela kubo uma behlangana nezinkinga noma nabangani abangathembekile?*

**1.7 Do you think other parents would want their daughters to participate in the groups?**<br><br>***Ingabe uca ukuthi abanye abazali bangafuna ukuthi amadodakazi abo abambe iqhaza kuleliqembu?***	• What some of the reasons that parents or caregivers might want their daughters to participate? / *Yiziphi ezinye izizathu zokuthi abazali noma abanakekeli okungenzeka bafune amadodakazi abo abambe iqhaza?*<br>• What some of the reasons that parents or caregivers might not let their daughters participate? / *Yiziphi ezinye izizathu zokuthi abazali noma abanakekeli okungenzeka bangafuni ukuthi amadodakazi abo abambe iqhaza?*<br>• Probe for disabled AGYW and migrant AGYW/ *Buzisisa abakhubazekile kwiAGYW kanye nabokufika kwiAGYW*
**1.5 Are girls and young women facing any other obstacles or challenges to participating in the groups?**<br><br>***Ingabe amantombazane kanye nabesifazane abasebancane babhekene nanoma iziphi ezinye izithiyo noma izinqinamba ngokubamba iqhaza emaqenjini?***	• Are girls and young women facing any other obstacles or challenges with any of the information or activities they learn about in the program? / *Ingabe amantombazane kanye nabesifazane abasebancane babhekene nanoma iziphi ezinye izithiyo noma izinqinamba nganoma yiluphi ulwazi noma ngemisebenzi abayifundayo ohlelweni?*<br>• Probe for differences between disabled AGYW and migrant AGYW/ *Buzisisa abakhubazekile kwiAGYW kanye nabokufika kwiAGYW*
Access to Key Resources and Protection – All participants	
**Lead questions**	**Secondary questions and probes**
**2.1 Are there organizations in your community that provide assistance for families who come to South Africa from other countries?**<br><br>***Zikhona izinhlangano emphakathini wakho ezisiza imindeni ezeMzansi Afrika evela kwamanye amazwe?***	• Do you know of anyone who has received assistance from these organizations? / *Ukhona omaziyo owathola usizo kulezi zinhlangano?*<br>• Do these organizations help girls and young women? / *Ingabe lezinhlangano ziyawasiza amantombazane noma abesifazane abangcane?*<br>• If so, in what ways have they helped them, for example, do they help with providing ID documentation or papers? / *Uma kunjalo, ingabe babasiza ngayiphi indlela, isibonelo, ingabe bayasiza ngokunikeza amadokhumenti ama-ID noma amaphepha?*<br>• Are these organisations at school or in the community? / *Ingabe zikhona lezinhlangano ezikoleni noma emphakathini?*<br>• Probe for disabled AGYW and migrant AGYW / *Buzisisa abakhubazekile kwiAGYW kanye nabokufika kwiAGYW*

**2.2. What kinds of health and HIV services do girls and young women use in your community?**<br><br>***Hlobo luni lwezinsiza lwezempilo kanye nesandulela ngculasi olusetshenziswa abantu besifazane abangcane kanye namantombazane emphakathini wakho?***	• Are these services used by young girls in your community? / *Ingabe lezinsiza zisetshenziswa abasefazane abancane emphakathini wakho?*<br>• Probe for migrant girls / *Buzisisa abesifazane bokufika*<br>• Probe for disabled girls / *Buzisisa abesifazane abakhubazekile*
**2.3. Before joining the program, did you know about HIV or family planning services?**<br><br>***Ngaphambi kokungenela loluhlelo ingabe wawu nalo ulwazi ngezinhlelo ngesandulela ngculazi noma izinsiza zokuhlela umndeni?***	• Did you or anyone you know access HIV or family planning services before the program? / *Ingabe wena noma ubani wakwazi ukuthola izinsiza zegciwane lesandulela ngculazi noma ezokuhlela umndeni ngaphambi kwaloluhlelo?*<br>• Has the program helped you and other young women connect with services, such as HIV testing, family planning, or other sexual and reproductive health services? / *Ingabe loluhlelo lusize ukuthi wena kanye nabanye abesifazane abancane ukuthi nixhumane nezinsiza, njengokuhlolela igciwane lesandulela ngculaza, ukuhlela umndeni, noma ezinye izinsiza zokuzalanisa kwezempilo. Noma ezinye izinsiza zezempilo zocansi nezokuzalanisa?*<br>• Probe for disabled AGYW and migrant AGYW / *Buzisisa abakhubazekile kwiAGYW kanye nabokufika kwiAGYW*
**2.4 Are most girls and young women in your community in school?**<br><br>***Ingabe iningi lamantombazane noma abesifazane abancane bayafunda emphakathini wakho?***	• Are young women able to attend their classes regularly? / *Ingabe abesifazane abancane bayakwazi ukuya ezikoleni zabo njalo njalo?*<br>• What difficulties do they have attending classes? / *Yiziphi izinkinga abahlangabezana nazo uma beya emakilasini?*<br>• What helps them to attend classes? / *Yini ebasiza ukuba bafunde amakilasi?*<br>• Probe for disabled AGYW and migrant AGYW / *Buzisisa abakhubazekile kwiAGYW kanye nabokufika kwiAGYW*
**2.5 Do girls and young women participating in this program attend school regularly?**<br><br>***Ingabe amantombazane kanye nabesifazane abasebancane ababambe iqhaza kuloluhlelo bayaya esikoleni ngokuvamile?***	• Has the program helped girls and young women feel motivated or excited about school and homework? / *Ingabe uhlelo luwasizile amantombazane nabesifazane abasebancane bazizwe bekhuthazekile noma bejabulile ngesikole kanye nomsebenzi wesikole wasekhaya?*

**2.6 Has the program (INSERT NAME OF PROGRAM) helped girls and young women connect with organizations in your community that are set up to provide support and help for them?**<br><br>***Ingabe uhlelo luwasizile amantombazane nabesifazane abasebancane ngokuhlangana nezinhlangano emphakathini wakho ezakhelwe ukunikezela ukweseka nokusiza bona?***	• Have they provided services or assistance that have been helpful to them? / *Ingabe banikezelile ngezinsiza noma usizo olube usizo kubona?*<br>• Are they having difficulties accessing services or assistance? / *Ingabe banobunzima ngokufinyilela ezinsizeni noma usizo?*<br>• How could the program assist with these difficulties? / *Uhlelo lungasiza kanjani ngalezi zinqinamba/ ngalobunzima?*
**2.7 Have you seen changes in AGYW's ability to access health and other services in the community?**<br><br>***Ingabe ulibonile ushintsho emantombazaneni nakubesifazane abasebancane ekukhoneni ukufinyelela kwezezimpilo kanye nezinye izinsiza emphakathini?***	• Does the program help girls and young women connect with new health and HIV services, such as HIV testing? / *Ingabe uhlelo luyawasiza amantombazane kanye nabesifazane abasebancane ngokufinyelela kwezezempilo ezintsha kanye nezinsiza zegciwane lesandulela ngculaza, njengokuhlola igciwane lesandulela ingculaza?*<br>• Are girls and young women accessing any help or support from the South African government as a result of the program, and in what forms of assistance if so? / *Ingabe lukhona noma yiluphi usizo olutholwa amantombazane nabesifazane abasebancane noma usizo noma ukusekwa kuhulumeni wase Ningizimu Afrika niengomphumela wohlelo sizo luni abaluthola, uma kunjalo?*<br>• Are they having difficulties accessing services or assistance? / *Ingabe banobunzima ngokufinyelela ezinsizeni noma usizo?*<br>• How could the program assist with these difficulties?/ *Uhlelo lungasiza kanjani ngalobunzima?*
**2.8 Have you seen changes in girls and young women's skills and self-confidence since they started to participate in the program?**<br><br>***Ingabe uke walubona ushintso ngamakhono kanye nokuzethemba bona emantombazaneni kanye nakubesifazane abasebancane kusukela ekuqaleni kwabo ukubamba iqhaza ohlelweni?***	• Are there ways that their skills could prepare them to avoid or speak up against discrimination or bullying? / *Zikhona izindlela zokuthi amakhono abo angakwazi ukubalungiselela ukugwema noma ukuba bakhulume ngokumelana nokubandlululwa noma noxhashazwa?*<br>• Are there ways that their skills could prepare them to avoid acts of xenophobia (as relevant)? / *Ingabe zikhona izindlela ezingenza ukuthi amakhono abo abalungiselele ukugwema izenzo zokucwasana ngobuzwe (ngokufaneleka)?*<br>• Are there any skills the program should focus on that could help AGYW in these situations? / *Ingabe akhona noma imaphi amakhono uhlelo okumele lugxile kuwo angakwazi ukusiza amantombazane nabesifazane abasebancane babhekane nalezizimo?*

**Conclusion**<br>*We are almost finished. I just have one more question before we end/*<br>*Sesiyaqeda.Nginombuzo owodwa ngaphambi kokuthi siqede*
What are your most important needs that still have not been sufficiently addressed?<br>*Do you have any questions for me about anything we have discussed? Is there anything else you would like to share?/*<br>*Iziphi izidingo zakho ezibalulekile kakhulu ezingakabhekelelwa ngokwanele?*<br>*Ingabe ukhona onemibuzo kimi nganoma yini esixoxe ngayo? Ingabe kukhona okunye ongathanda ukwabelana ngakho?*

This is the end of the focus group discussion. Thank you so much for sharing your ideas with us. Do you have any questions, or is there anything that you would like to add before we end? If you have further thoughts about any of the issues we discussed today, please call Dr. Mags Beksinska, the South African Principal Investigator of the study, whose details are on the information sheet and consent form that you have been given./

Lokhu sekungukuphela kwengxoxo yeqembu.Ngiyabonga ngokwabelana ngemibono yenu nathi. Ingabe unemibuzo, noma Ngabe kukhona ofuna ukukwengeza ngaphandle ngaphambi kokuqeda? Uma uneminye imibono mayelana nanoma yiziphi izinkinga esixoxe ngazo namhlanje, sicela ufonele uDokotela Mags Beksinska, umphenyi oyinhloko yaseningizimu Afrika locwaningo, imininingwane yakhe etholakala kwipheshana lolwazi noma imvume nolwazi onikwezwe lona.
